# A systematic review and meta-analysis protocol of clinical characteristics and prognostic significance of mammalian target of rapamycin for gastric cancer patients

**DOI:** 10.1097/MD.0000000000021138

**Published:** 2020-08-07

**Authors:** Hua Wang, Juan Li

**Affiliations:** a Department of Hepatobiliary Surgery, The Second Hospital of Lanzhou City; b Department of Pathology, The First Hospital of Lanzhou City, Lanzhou, China.

**Keywords:** gastric cancer, meta-analysis, mammalian target of rapamycin, overall survival

## Abstract

**Background:**

The high morbidity and mortality of Gastric cancer (GC) is seriously endangered human health. Owing to the low rate of early diagnosis and human body can resistant to the anti-tumor drugs, so an early diagnostic biology marker is essential. However, recently studies indicated that Mammalian target of rapamycin (mTOR) is usually frequently deregulated in many cancers, especially in GC. And the efficacy of mTOR inhibitor was promising in a phase II clinical trial which could inhibited the proliferation of GC cells and delayed tumor progression. Therefore, mTOR were identified as a potential prognosis biomarker for GC, and its inhibitor will be promising in anti-GC therapy. The main aim of this systematic review and meta-analysis is to investigate the relationships between the expression level and prognostic value of mTOR in patients with GC.

**Methods:**

Four electronic databases were systematically searched as follow: the PubMed, EMbase, Web of Science databases, and the Cochrane Library. All the data will be extracted by independent researchers from the eligible studies with the inclusion and exclusion criteria. And the data will be analyzed through STATA 12.0 software.

**Results and conclusion:**

This meta-analysis indicated that overexpressed mTOR was significantly in predicting a poorer prognosis for GC patients. The expression level of mTOR should be considered as a potential independent prognostic predictor for GC patients.

**Protocol registration number:**

CRD42020159690.

## 1 Introduction

Gastric cancer (GC) is the fourth most prevalent malignancies and the second leading cause of cancer death over the world,^[1]^ and which is more seriously in China.^[2]^ Owing to the high morbidity and mortality, GC has become a big threat to human health. According to the National Comprehensive Cancer Network (NCCN), surgery is the standard treatment for GC patients, and the chemotherapy, radiation therapy, and is an adjuvant treatment.^[3]^ Prognosis of patients is mostly depended on the GC stage, and the majorities are diagnosed as advanced or unresectable GC at the initial diagnosis. However, the 5-year overall survival (OS) of early GC patients after curative resection is more than 90%, while the advanced patients is only 10% to 30%.^[4,5]^ Owing to the low rate of early diagnosis, identifying a prognostic marker for GC patients which may be also a potential treatment target is a task which brooks no delay.

Recently, the advance in understanding of genetic and epigenetic biology in GC contribute to developing and exploring novel promising therapeutic strategies and drugs to control GC growth and metastasis, and also could be used in improving the outcomes.^[6,7]^ The evidence of recent studies indicated that mammalian target of rapamycin (mTOR) is a serine/threonine kinase that gets inputs from the amino acids, nutrients, growth factor, and environmental cues to regulate varieties of fundamental cellular processes, include protein synthesis, metabolism, growth, aging, regeneration, autophagy.^[8]^ And a large number of studies have shown that it plays an important role in regulate GC cell growth and proliferation, and abnormal regulation of mTOR signaling pathway is closely related to GC cell proliferation.^[9,10]^ Further, both of the monotherapy of mTOR inhibitor and the combination with chemotherapy exert a promising efficacy in treatment of GC.^[11]^ Therefore, the mTOR could be a potential important biomarker in prediction of the prognosis of GC patients. The aim of our systematic review and meta-analysis is to evaluate the overexpressed mTOR in GC patients is related to a poorer prognosis. The expression level of mTOR should be considered as a potential independent prognostic predictor for GC patients.

## 2 Methods

### 2.1 Registration

Our systematic review protocol was performed with accordance to the preferred reporting items for systematic review and meta-analysis protocols extension statement.^[14]^ The registration number of our protocol is CRD42020159690, Which has been registered on the international prospective register of systematic review.

### 2.2 Ethics and dissemination

#### 2.2.1 Ethics issues

This systematic review does not require ethics approval or obtaining informed consent. On account of this systematic review is a secondary analysis based on previously published original data, and is not need direct contact with the individual patients.

#### 2.2.2 Publication plan

This systematic review will be published in a peer-reviewed journal and disseminated through conference posters or abstracts.

### 2.3 Inclusion criteria

#### 2.3.1 Types of studies

Studies reported the hazard ratio (HR) value and the corresponding 95% confidence interval for OS directly, or can be obtain from the original text or calculation indirectly;

#### 2.3.2 Types of participants

GC patient was diagnosed by histopathological confirmation without age, gender and racial limitations (diagnosed and classified as proposed by NCCN guideline^[12]^);

#### 2.3.3 Type of outcomes

The primary outcomes are the survival rates and OS. The secondary outcomes are baseline characteristics of GC patients.

### 2.4 Information source

The PubMed, EMbase, Web of Science databases, and the Cochrane Library were systematically searched for identifying the potential eligible published studies. The following keywords were conducted for the retrieval: “Gastric Cancer”, “Gastric Neoplasm”, “Cancer of Stomach”, “Stomach Cancer”, “Stomach neoplasm”, “neoplasm of the Stomach”, “mTOR”, and “mechanistic target of rapamycin”. The deadline of search time is 30^th^ November 2019. Further, the references of included articles and relevant systematic reviews and meta-analysis will be also searched to identify other additional studies.

### 2.5 Data collection and analysis

#### 2.5.1 Data management

ENDNOTE X7 (Thompson Reuters, CA) was applied to manage literature search records. Before the literature selection, a pilot-test will be conducted between the reviewers to ensure high inter-rater reliability.

#### 2.5.2 Selection process

In accordance with the formulated search strategy, two independent reviewers will screen the title and abstract retrieved studies. If the title and abstract screening are passed, the potentially qualified study will be re-evaluated by retrieving the full text. If there is any objection, we still need a third reviewer. According to the preferred reporting items for systematic review and meta-analysis protocols guidelines the study selection process will be revealed in a flow diagram.^[13]^

#### 2.5.3 Data collection process

A standard data abstraction form which would be used to extract data was created in Microsoft Excel 2010 (Microsoft Corp, Redmond, WA, www.microsoft.com). Two reviewers will complete data extraction, and will check the consistency and accuracy of all of the extracted data. Any disagreements will be resolved by discussion with a third reviewer. The data extracted were listed as follows:

(1) outcome indicator OS: HR and 95% confidence interval can be extracted directly in the original text, otherwise, we need to calculate the outcome indicators from K-M curve indirectly through Engauge Digitizer 4.1 software;^[14]^
(2) baseline characteristic include author's first name, publication date(year), region, sample size, patients’ gender, age, TNM stage and treatment situation.

### 2.6 Quality of evidence assessment

According to the quality assessment tool for the prognostic study,^[15]^ each included studies was evaluated by 3 independent researchers with “Yes”, “partly”, “no” or “unsure” respectively in 6 aspects which were listed as follow: “study participation”, “study attrition”, “prognostic factor measurement”, “outcome measurement”, “confounding measurement and account” and “analysis”

### 2.7 Risk of bias individual studies

Cochrane Handbook version 5.1.0^[16]^ which assess 7 specific domains: sequence generation (selection bias), allocation concealment (selection bias), blinding of participants and personnel (performance bias and detection bias), incomplete outcome data (attrition bias), selective reporting (reporting bias), and other bias and the risk of bias of all included RCTs will be estimated using it. Based on criteria of the risk of bias judgment,^[17]^ we will evaluate methodological quality as low risk, high risk, or unclear risk of bias.

The risk of bias of included non-randomized studies will be evaluated according to the tool for assessing risk of bias in non-randomized studies of interventions (ROBINS-I),^[18]^ including bias due to confounding (pre-intervention), bias in selection of participants into the study (pre-intervention), bias in classification of interventions (at intervention), bias due to deviations from intended interventions (post-intervention), bias due to missing data (post-intervention), bias in measurement of outcomes (post-intervention), bias in selection of the reported result (post-intervention), and overall risk of bias. We will evaluate risk of bias as low, moderate, serious, critical risk of bias, and no information.

Two reviewers will complete the easement of risk of bias independently. The conflicts will be resolved by a third reviewer.

### 2.8 Statistics analysis

Excel 2010 will be used to summarize and show data of all the included studies and their major characteristics related to the aim of this systematic review and meta-analysis. The pooled HR and its 95% CI were used to evaluate the relationship between the expression level of mTOR and OS. Heterogeneity among the studies was evaluated by *X*^2^ -based *Q*-test: *I*^2^ > 50% which suggested the statistical heterogeneity is significant, and the random-effect model (the Mantel-Haenszel method) was selected, otherwise, the fixed-effect model was adopted in this meta-analysis.

### 2.9 Other analyses

#### 2.9.1 Subgroup and sensitivity analyses

The subgroup analysis was designed by GC patent's age, gender, TNM stage, tumor size, lymph node metastasis, invasive depth, and histological grade, which Will be used to find the possible sources on account of a possibility of significant heterogeneity or inconsistency.

#### 2.9.2 Publication bias

STATA V.12.0 software (Stata Corporation, CollegeStation, Texas) will be performed to draw a comparison-adjusted funnel plot to identify whether there will be a small sample effect. Galbraith plot analysis and sensitive test will be conducted to test the heterogeneity among included studies and ensure the stability of pooled results, and the Egger and Begg Test will be used to evaluate the potential publication bias.^[19]^

## 3 Disscusion

It is anticipated that the results of this research will indicating whether mTOR have diagnostic and/or prognostic utility.

## Author contributions

WH and LJ planed and designed the research; WH, LJ and MTY tested the feasibility of the study; WH wrote the manuscript. All authors approved the final version of the manuscript.

**Conceptualization:** Hua Wang, Juan Li.

**Data curation:** Taiyu Mi.

**Investigation:** Taiyu Mi.

**Methodology:** Juan Li.

**Project administration:** Juan Li, Hua Wan.

**Resources:** Hua Wan, Taiyu Mi.

**Software:** Taiyu Mi.

**Supervision:** Juan Li.
